# Training for the future NHS: training junior doctors in the United Kingdom within the 48-hour European working time directive

**DOI:** 10.1186/1472-6920-14-S1-S12

**Published:** 2014-12-11

**Authors:** Shreelatta T Datta, Sally J Davies

**Affiliations:** 1 King's College Hospital, NHS Foundation Trust, London, UK; 2 University Hospital of Wales and Vale University Health Board, Cardiff, UK

## Abstract

Since August 2009, the National Health Service of the United Kingdom has faced the challenge of delivering training for junior doctors within a 48-hour working week, as stipulated by the European Working Time Directive and legislated in the UK by the Working Time Regulations 1998. Since that time, widespread concern has been expressed about the impact of restricted duty hours on the quality of postgraduate medical training in the UK, particularly in the “craft” specialties – that is, those disciplines in which trainees develop practical skills that are best learned through direct experience with patients. At the same time, specialist training in the UK has experienced considerable change since 2007 with the introduction of competency-based specialty curricula, workplace-based assessment, and the annual review of competency progression. The challenges presented by the reduction of duty hours include increased pressure on doctors-in-training to provide service during evening and overnight hours, reduced interaction with supervisors, and reduced opportunities for learning. This paper explores these challenges and proposes potential responses with respect to the reorganization of training and service provision.

## Introduction

The European Working Time Directive (EWTD), issued in 1993 by the European Union with the intention of protecting the health and safety of workers, sets out minimum requirements in relation to work hours, rest periods, and annual leave. It was enacted in the United Kingdom as the Working Time Regulations 1998 (WTR), with subsequent amendments between 2001 and 2007 [1-7]. To give the National Health Service (NHS) time to adjust, junior doctors were initially exempt from these regulations. In August 2004, the directive was extended to include junior doctors, whose working hours were gradually reduced, reaching an average of 48 per week in August 2009.

In 1991, doctors’ representatives, the medical royal colleges, NHS managers, and the government had agreed to the New Deal, a package of measures designed to improve the working conditions of junior doctors; this agreement reduced the maximum number of hours worked by junior doctors on full shifts to 56 per week [8]. The New Deal is not superseded by the WTR; both are applicable, and where requirements vary the more stringent criteria must be followed (see Table 1).

**Table 1:** Stipulations of the WTR and New Deal

	Working Time Regulations	New Deal
Working hours	• 48-hour weekly average over defined period. Working hours include: – Any period spent working – Relevant training time – All compulsory resident hours	• 56-hour maximum weekly average. Working hours include: – Time spent on duty carrying out tasks – Study leave/training • Maximum 13 continuous duty days

Rest periods	• 11 consecutive hours in each 24-hour period • Uninterrupted 24-hour period of rest in each 7-day period (or 48 hours in 14 days) • Minimum 20-minute break every 6 hours worked • Compensatory rest	• Dependent on shift pattern • 30-minute break every 4 hours on more than 75% of occasions

Opting out	• Individual doctor choice • In writing	• Can do no more than average of 56 hours of actual work a week

Annual leave	• Minimum 4 weeks paid annual leave	• Annual leave dependent on years worked

Monitoring	• Average working hours of individual doctor over 26-week reference period	• Minimum 2-week monitoring of rota compliance twice a year

Sanctions for non-compliance	• Improvement notice • Prohibition notice • Fine (£5,000/employee/week) • Imprisonment of responsible authority	• Grievance raised • Higher salary multiplier

The traditional experiential model of training in the United Kingdom had relied on trainees spending long hours delivering service across the NHS, developing their skills and knowledge in an often unstructured manner. As we will discuss in the following section, the EWTD has challenged this model, the working patterns of hospital doctors, and the organization of service provision by health care employers.

### Impact of the European Working Time Directive on training

There is early evidence that compliance with the EWTD duty hour restrictions has had no adverse effects on patient safety or quality of care [9] and may have reduced the incidence of medical errors [10]. The introduction of competency-based specialty curricula, workplace-based assessment, and the annual review of competency progression (ARCP) has resulted in significant changes for doctors entering specialty training from 2007 onward [11]. By the same token, given the limited time that has elapsed since the introduction of the 48-hour average in August 2009, the full impact on validated training outcomes is not yet known. Assessment of outcome measures is also complicated by variables such as the changes in the structure of training, new ways of working, and increased pressure on junior doctors to cover gaps in the rota to provide services [12]. In addition, the implementation of recommendations from the National Confidential Enquiry into Patient Outcome and Death (NCEPOD) has reduced out-of-hours (evening, nighttime, and weekend) training opportunities [13], which typically strengthen independent decision-making and practical ability.

There is increasing pressure from employing organizations to maximize service provision at the expense of learning opportunities. Covering service needs with doctors-in-training means that these junior doctors must work proportionately more hours outside the daytime hours. Adding extra doctors to training-grade rotas to comply with EWTD requirements further “dilutes” the available experience as the number of patient interactions per doctor decreases.

The challenge is to provide high-quality training opportunities, maximize daytime working hours, raise the standards of specialty training, and produce specialists who are competent and confident.

### Obligations under the Working Time Regulations

Under the WTR, all doctors in training must work no more than 48 hours a week, averaged over a reference period of 26 weeks (Table 1). In the SiMAP ruling, which pertained to a case initiated in Spain by the Sindicato de Médicos de Asistencia Pública, the European Court of Justice defined all of the time during which a doctor is required to be present at the site where care is provided as working hours. The Jaeger judgment, which pertained to a similar case in Germany, confirmed this ruling, made it more explicit that this included time when the doctor was able to sleep while on-call in the hospital, and stipulated provisions for compensatory rest when normal rest requirements could not be met.

The reduction in hours has been widely perceived as having a negative impact on training [14,15]. In a survey of consultant physicians in the United Kingdom, 83% of respondents indicated that they felt the quality of training had deteriorated after the implementation of the EWTD [16]. Concerns have been raised by professional colleges that doctors who complete training under the new duty hour restrictions are less experienced, less confident, and have fewer skills than their counterparts who were trained before the regulations. However, no objective evidence has been provided to support these claims. In some specialties, up to 80% of trainees surveyed reported that the EWTD resulted in fewer training opportunities, and over half said that their job satisfaction had been reduced [17]. Lost training opportunities reported in the surveys pertained to operating theatre experience, handovers, clinics, ward rounds, audit, research, and attendance at conferences [15].

Patient care, particularly in out-of-hours periods, relies on a shift system in which the available workforce provides cover. Although, on paper, rotas are reported as EWTD-compliant, in practice they frequently have gaps [8]. These gaps are most likely to occur in the evenings or at night, when supervision is minimal and there are fewer training opportunities. Trainees are moved at short notice from their daytime, often elective, training commitments to fill these service gaps, thus sacrificing planned, supervised training opportunities.

### Potential solutions in the United Kingdom

A range of adaptations and innovations might be made to address the challenges posed by the reduction of resident duty hours. In this section we outline the advantages and drawbacks of nine potential solutions. A summary is provided in Table 2.

**Table 2:** Potential solutions and impact

Strategy	Implementation and impact
Adjusting the length of training	• Implemented locally in some specialties only in view of the funding and resources required • Positive feedback where implemented

Redesigning rotas	• Increased anti-social working hours • Non-resident on-call has been implemented in some specialties • Trainees have opted out of the EWTD because of rota gaps in some acute care specialties

Using operating lists dedicated to training	• Popular with trainees and trainers alike • Limited by employer productivity targets

Setting targets for number of each procedure performed	• Patchy implementation in some specialties • Targets are limited by individual learning paces and availability of the correct patient population

Using simulation technology for training	• Advocated by the Department of Health • Limited availability locally because of cost

Reconfiguing services	• Hospital at Night has successfully encouraged multidisciplinary work and cross-specialty cover • Training in recognized centres only: not popular

Including periods of supernumerary training	• In place in General Practice training programs but not generally available • Limited by resources

Increasing consultant numbers	• Gradually under way in some acute specialties such as Obstetrics • Limited by financial constraints in NHS

Providing adequate educational governance	• Standards set by the GMC with regular trainee questionnaires and visits to specialty training schemes • Educator roles to be encouraged and recognized

### 1. Adjusting the length of training

One possible response to the reduced number of hours available for training per week is to ensure that the length of training is premised on the attainment of competencies. This could be achieved either by prospectively lengthening the time for training, or by making the duration of training more flexible. Lengthening training duration is currently being considered by the Royal College of General Practitioners. Arguments against this approach include lack of evidence of educational benefits and financial burden to the employer [18].

Less than full-time training (LTFT) is available in the United Kingdom for trainees with health concerns or familial responsibilities as caregivers; therefore, some trainees work proportionately fewer hours and take longer to achieve the required competencies than their peers. There is no evidence that these doctors are less competent at the completion of their training than those who have trained within the usual time frame [19]. The ARCP enables trainees who are not achieving the required competency standards to extend their training for the purpose of targeted remediation [11].

### 2. Redesigning rotas

Changes to achieve EWTD compliance require more doctors per weekly rota, thus increasing the pressure on junior doctors to work fewer daytime hours in favour of providing coverage during overnight shifts. This has had the effect of reducing trainer–trainee interactions, impeding continuity of care, and increasing the number of handovers required [20]. Responses to a survey of junior doctors conducted by the BMA in 2010 included the following:

Working a shift pattern means continuity of patient care and time spent on the ward has been reduced … reducing not only learning opportunities but also the ability to learn from/about the progression of a disease.

With regards to my health and safety and quality of life … although the hours have reduced the number of unsocial hours has increased [15].

The effect of the EWTD varies at different levels of training. For example, senior trainees are often unavailable for more specialized elective training opportunities when they cover a generalist emergency rota [21], and junior trainees may feel exposed without sufficient clinical supervision out of hours [12]. There are two potential ways to adjust rotas to compensate for the reduction of available resident duty hours:

• Non-residents working for senior trainees. One way to maximize the training available is to ensure that only necessary doctors are resident when on call. This would allow trainees to come to the hospital after their on-call shift to take up daytime educational opportunities. This system has been successfully implemented locally by many of the surgical specialties, although limiting factors include the variable intensity of the on-call shift.

• Opting out of the EWTD. Although an individual doctor can voluntarily “opt out” of the limit on working hours, junior doctors are contractually limited under the New Deal to a maximum average of 56 hours of actual work a week. In addition, junior doctors cannot opt out of the rest requirements stipulated by the WTR (Table 1). In a survey of Obstetrics and Gynaecology trainees, 74% of respondents were found to work voluntarily on their days off to take advantage of training opportunities they were missing [22].

### 3. Using operating lists dedicated to training

Trainees often spend time assisting with complex procedures that are unsuitable for their stage of training. Operating lists in which the trainee works as a first operator rather than an assistant in procedures that trainees need to perfect should be encouraged. Dedicated training lists are popular with trainees and trainers but can take longer, thus increasing pressure on theatre time and the organization’s need to achieve efficiency targets.

### 4. Setting targets for number of each procedure performed

Competency-based training has led to a move away from target numbers for certain procedures [23]. However, setting benchmarked targets for procedures performed would allow trainees to know whether they are getting appropriate experience in a particular training post. This may identify the need to extend training if a post fails to provide adequate experience to develop competence.

### 5. Using simulation technology for training

Where they are available, simulation facilities are accessible at all times of the day and night and allow trainees to improve their skills with educational supervision as needed [24]. Trainees can identify a weakness and develop their technique immediately under supervision, without the risk of harming a patient. Identified individual training needs can be targeted in the skills laboratory.

### 6. Reconfiguring services

Organizations that have designed new ways to work and train have achieved benefits such as safer clinical service for patients and enhanced quality of training. The national “Hospital at Night” initiative allows trainees to maximize daytime working hours while providing safe patient care [25]. Hospital at Night is a clinically driven, patient-focused change program that uses a multi-professional and multi-speciality approach to delivering care at night and out of hours [26].

Multidisciplinary teamwork provides valuable training opportunities. This is particularly useful at handovers, which can be effective learning experiences when they are supervised by senior staff, preferably consultants. Training should be delivered in a service environment with appropriate, accredited consultant supervision both during daytime periods and out of hours [27].

### 7. Including periods of supernumerary training

Periods of supernumerary training could be included within training programs, thus allowing for dedicated training periods interspersed with service experience. In the United Kingdom, this occurs in the General Practice specialty training. The cost and service implications of dedicated periods of supernumerary training in all specialties have yet to be considered.

### 8. Increasing consultant numbers

Consultant-led care can result in efficiency savings and enhanced patient safety [28]. Given that the numbers of doctors-in-training is controlled and the number of hours worked is reduced, it is important that specialist capacity be expanded to manage the service, provide time for high-quality training, and potentially develop new ways to deliver care. It is not feasible to provide training in all hospitals, as currently occurs in the United Kingdom. Organizations that offer excellent training opportunities should be encouraged to continue to do so, and consultants who provide high-quality education should be supported and rewarded.

### 9. Providing adequate educational governance

Health organizations that provide physician training should put educational governance high on their agenda and systematically review outcomes. A positive training culture should be rewarded. The General Medical Council has published standards for training and monitoring that provide the basis for quality assurance for commissioners [27]. Annual UK-wide trainee questionnaires, regular inspection visits, and monitoring of the ARCP reports and of outcomes of specialist training should provide robust information about the quality of training under the EWTD.

## Conclusion

The implementation of the EWTD in the United Kingdom since August 2009 has significantly changed the working pattern of junior doctors. The impact on training remains difficult to quantify. The key challenge is to find ways to protect educational opportunities in a service under pressure to provide 24-hour health care by doctors-in-training. The reduction in hours has led to reduced daytime training and more evening and nighttime shifts, when training potential is reduced. Solutions will need to focus on the reorganization of training and service delivery. The requirement for specialist-led care and designated training centres also needs to be addressed. Periods of supernumerary training should be considered, although the implications for service must be taken into account. Improved educational governance and monitoring of the outcomes of training by the regulator can inform reorganization and help to ensure that training is provided by the most suitable personnel.

Because trainees in the NHS learn in a service-based environment, learning opportunities in every clinical situation must be optimized. To achieve this objective, fundamental changes to the delivery of training and service are required. The innovations that improve training are limited by a perceived slow rate of adoption by the local NHS organizations that deliver training. Although some of the solutions suggested in this paper will require additional resources and funding, this should be regarded as a prudent investment in high-quality care and the enhancement of patient safety.

## Authors’ contributions

STD produced the first draft. This draft was edited by SD, and then the paper was finalized by both authors.

## Competing interests

ST Datta is a UK junior doctor, Chair of the North European Doctors Association, and Past Chair of the British Medical Association Junior Doctors Committee. SJ Davies is a Sub Dean of Post-graduate Medical Education and President elect of the Medical Women’s Federation.

## Declarations

Resources and secretariat support for this project was provided by the Royal College.

This article has been published as part of *BMC Medical Education* Volume 14 Supplement 1, 2014: Resident duty hours across borders: an international perspective. The full contents of the supplement are available online at http://www.biomedcentral.com/bmcmededuc/supplements/14/S1. This article was submitted peer reviewed in 2012. Final acceptance for publication as part of this supplement was in 2014.

Publication of this supplement was supported and funded by the Royal College of Physicians and Surgeons of Canada. The funding agency played no role in the design, in the collection, analysis and interpretation of data; in the writing of the manuscripts; and in the decision to submit the manuscript for publication. The articles have been through the journal's standard peer review process for supplements. The Supplement Editors declare that they have no competing interests.
